# Risk factors associated with prolonged stay in the post-anesthesia care unit: a case-control study

**DOI:** 10.3389/fmed.2026.1906741

**Published:** 2026-09-17

**Authors:** Zhen Xue, Na Zhang, Linnian Liu, Xin Wei

**Affiliations:** 1 Department of Anesthesiology, Anhui Aier Eye Hospital, Hefei, Anhui, China; 2 Department of Anesthesiology, The First Affiliated Hospital of USTC, Division of Life Sciences and Medicine, University of Science and Technology of China, Hefei, Anhui, China; 3 Department of Nursing, Anhui Aier Eye Hospital, Hefei, Anhui, China

**Keywords:** general anesthesia, nomogram, post-anesthesia care unit, prealbumin, risk factors

## Abstract

**Background:**

Prolonged length of stay in Post-Anesthesia Care Unit (PLOS in PACU) reduces PACU bed turnover rates, increases healthcare costs, and lowers patient and surgeon satisfaction. This study aims to identify additional factors associated with prolonged PACU stay and to develop a nomogram for individualized risk assessment.

**Methods:**

This retrospective study included 69,123 adult patients who were transferred to PACU after general anesthesia from January 2022 to January 2024. PLOS was defined as PACU stay > 2 hours; non-PLOS controls were matched 3:1 by operative date (±1 month), age (±1 year), and gender. A predictive model was developed using LASSO regression for variable selection and multivariable conditional logistic regression, then presented as a nomogram.

**Results:**

Among 68,178 eligible patients, 210 subjects were identified as PLOS in PACU. Multivariable conditional logistic regression showed that the potential risk factors were emergency surgery (OR 7.678, 95% CI 2.606-22.618, *P* < 0.001), increased intraoperative blood loss (OR 1.002, 95% CI 1.001-1.004, *P* = 0.005), longer operative time (OR 1.004, 95% CI 1.001-1.008, *P* = 0.011), cerebrovascular diseases (OR 2.248, 95% CI 1.162-4.346, *P* = 0.016) and major surgery (OR 4.954, 95% CI 1.801-13.624, *P* = 0.002). The optimism-corrected AUC of 0.825 (95% CL 0.801–0.849). A higher preoperative prealbumin level (OR 0.990, 95% CI 0.986-0.994, *P* < 0.001) was inversely associated with the risk of PLOS in PACU.

**Conclusions:**

This study found that a higher preoperative prealbumin level was associated with a lower risk of prolonged PACU stay, possibly reflecting patients’ preoperative nutritional status, systemic inflammation, chronic illness, or frailty. A nomogram was constructed, though its single-center and exploratory nature requires external validation.

## 1 Introduction

The Post-Anesthetic Care Unit (PACU) is a specialized area designed to provide centralized monitoring and care for postoperative patients, and serves as a unit to boost workflow and turnover efficacy (1). Prolonged length of stay in Post-Anesthesia Care Unit (PLOS in PACU) reduces PACU bed turnover rates, increases healthcare costs, and lowers patient and surgeon satisfaction (2).

Definitions of PLOS in PACU vary widely. Both the Chinese Society of Anesthesiologists and the Australian Council on Healthcare Standards (ACHS) define PLOS as a PACU stay exceeding 120 minutes (3). Two Chinese studies used this threshold, the incidence in one study was 1.38% (4, 5). Using the same uniform definition, one study from the United Arab Emirates (UAE) reported 1.8%, while a study from Pakistan found 8.1% (6, 7). A study from Saudi Arabia applied a threshold of 90 minutes and reported an incidence of 1.19% (1). Other researchers have adopted the 75th percentile as the threshold for defining prolonged PACU stay. Two U.S. studies focusing on ambulatory surgery followed this statistical method and identified different cut-off values. One study reported a 25.39% incidence of prolonged PACU stays in its development set using a cut-off of more than 100 minutes (8). The other reported a 5.31% incidence with a three-hour cut-off under the same standard (9). One Chinese study applied this same standard, setting the cut-off at ≥ 80 minutes and reporting an incidence of 24.9% within its development cohort (10). In Ethiopia, one study focusing on nonclinical factors defined PACU delay as patients who achieved an adequate Aldrete score but remained in the unit, with an incidence of 61.2% (2). These differences may arise from inconsistent definitions of prolonged PACU stay and variations in study design.

The cause of PLOS is classified as clinical and non-clinical factors. The clinical factors encompass the patients’ baseline condition, the complexity of the surgical intervention, and the anesthetic management (6, 11, 12). Previous studies have found clinical risk factors for prolonged PACU stay, such as advanced age, obesity, hypertension, asthma, female, ASA physical status III or higher, BMI < 21 kg/m^2^, obstructive sleep apnea, neurosurgical procedures, prolonged operative duration, intraoperative blood transfusion, surgical specialty, general anesthesia, extended anesthesia time, postoperative pain, shivering, delirium, postoperative ventilation, hemodynamically unstable, postoperative bleeding, and naloxone administration (1, 4, 6–11). Nonclinical factors include PACU bed availability, ward nurse unavailability, unclean recovery room, meal break, patient transport logistics, wait times for physician discharge orders, unavailability of beds in the special care areas ，and staffing levels (2, 6, 7, 13).Additionally, these delays also correlate with the hospital's surgical volume and the proportion of surgical categories within the hospital.

Several prediction models have been developed to identify patients at risk of prolonged length of stay in the PACU. Gabriel et al. built a logistic regression model for ambulatory surgery patients. The data were split chronologically into training and test sets, yielding AUCs of 0.754 and 0. 722.The model included only preoperative variables, which may be useful for risk stratification before surgery, but it did not incorporate intraoperative events or PACU complications. In addition, the lack of external validation across centers limits the generalizability of the results (8). Fang et al. constructed a LASSO-based logistic nomogram for 24,017 elective surgery patients, with C-indices of 0.773 in the training set and 0.757 in the validation set. The study had a large sample and included PACU complications such as shivering and delirium as predictors. However, its single-center retrospective design, lack of external validation, and exclusion of neurological, cardiac, and emergency procedures limit the generalizability of its findings (10). Zhang et al. derived a nomogram from a generalized linear model in a two-center cohort of 38,796 patients undergoing general anesthesia. Internal validation gave a C-index of 0.78, and external validation yielded an AUC of 0.70. This study had a large sample and included external validation, though performance declined in the external cohort. The model was built mainly on clinical variables; older age and neurosurgery carried higher weights, but non-clinical factors were not included (4). Tully et al. built machine learning models from 10,928 ambulatory surgery cases, applying SMOTE to address class imbalance during training. The top model's test-set AUC was 0.712. A simulation that reordered cases by predicted risk reduced after-hours PACU occupancy. The model was built on preoperative data only. Intraoperative factors and PACU events were not included, nor was external validation performed (9). Moreover, the above studies used different definitions for prolonged PACU stay.

Existing studies of prolonged PACU stay have been limited by incomplete variable coverage, insufficient confounder adjustment, inconsistent PLOS definitions, and lack of external validation. It remains unclear which clinical factors are potentially associated with PLOS after adjusting for confounders. In this study, we aimed to identify additional factors associated with PLOS after confounder adjustment and to develop a nomogram for individualized risk assessment.

## 2 Materials & methods

### 2.1 Study design

This was a retrospective, single-center, case-control study, which was conducted at the main surgical center of the First Affiliated Hospital of the University of Science & Technology of China (Anhui Provincial Hospital), where the majority of surgical procedures were orthopedic, general abdominal, and thoracic surgery.

### 2.2 Ethics statement

This study was approved by the Medical Research Ethics Committee of the First Affiliated Hospital of University of Science and Technology of China (approval no. 2025_RE_364; October 3, 2025), and informed consent was waived. All procedures followed the Declaration of Helsinki (2024 revision).

### 2.3 Study population

All data were collected from January 2022 to January 2024 through the hospital's electronic medical record. We included only adult patients transferred to the PACU after general anesthesia, as their perioperative complication profiles differ from those of children (14). Exclusion criteria included: 1) surgery was canceled after general anesthesia, as these patients had no surgical exposure; 2) non-intubated general anesthesia, since airway management and PACU recovery differ from intubated cases (15); 3) cardiovascular surgery, in which patients were routinely transferred directly to the cardiac intensive care unit after the procedure; 4) neurosurgery, because this population has established risk factors for delayed PACU recovery (4). We excluded them to explore other potential predictors; 5）patients who were transferred directly from the surgical ward to the ICU; 6）critical PACU records were missing, as essential variables required for outcome evaluation could not be retrieved from original medical charts.

### 2.4 Control

Patients were categorized into two groups based on PACU stay duration: the PLOS group (stay duration > 2 hours) and the non-PLOS group (stay duration ≤ 2 hours). With a case-control design, each patient in the PLOS group (due to anesthetic factors) was matched with 3 patients in the non-PLOS group based on the operative date (±1 month), sex, and age (±1 year).

### 2.5 Data sources and collection

The following information was collected using the hospital's electronic medical record system: 1) Patients’ baseline characteristics: age, sex, body mass index (BMI), history of comorbidities, American Society of Anesthesiologists (ASA) physical status classification, emergency/elective surgery, type of surgery, and surgical classification; 2) Preoperative laboratory values (most recently): prealbumin (PA), albumin (Alb), hemoglobin (Hb), alanine aminotransferase (ALT), aspartate aminotransferase (AST), serum creatinine(Scr), blood urea nitrogen (BUN), etc. 3) Intraoperative information: operative time (min), intraoperative blood loss (mL), time of surgery initiation (am/pm/night), etc. 4) PACU data: body temperature at admission to the PACU, total PACU stay duration, extubation time in PACU, the occurrence of nausea and/or vomiting, postoperative agitation, etc., (1, 2, 4, 6, 10, 11, 16).

### 2.6 Definition of variables

In this study, patients who stayed in PACU longer than 2 hours were defined as PLOS in PACU (4). Hypoxemia was defined as an oxygen saturation less than 90% while breathing room air (17). Admission to PACU hypothermia was defined as a core body temperature(tympanic temperature) below 36.0°C upon transferring to PACU (18). Delayed awakening was defined as failure to regain consciousness 90 minutes after the cessation of general anesthesia, along with an inability to respond appropriately to external stimuli and verbal commands (19). Hypertension was defined as a systolic blood pressure (BP) > 160 mmHg lasting for more than 5 minutes and/or an increase of ≥ 20% of the baseline value. Hypotension was defined as a systolic BP < 90 mmHg lasting for more than 5 minutes and/or a decrease of ≥ 20% from the baseline value (12). Arrhythmia was defined as any arrhythmia resulting in hemodynamic instability. Surgery was classified as minor, intermediate, or major. Minor surgeries (Grade I) include the removal of superficial skin masses, varicose veins, and breast nodules; skin debridement; and endoscopic procedures, such as hysteroscopy. Intermediate surgeries (Grade II) include procedures for gallstones, hepatic and renal cysts, thyroid cancer, wedge resection of the lung, and joint replacement. Major surgeries (Grade III) include treatment of liver, gallbladder, gastric, pancreatic, and renal cancers, as well as lobectomy of the lung (10).

In the present study, the following time periods were defined: morning (am) from 7:30 to 11:59, afternoon (pm) from 12:00 to 18:59, and night from 19:00 to 07:29 the next day. These definitions were applied to both the start time of surgery and the admission time to the PACU (20, 21). Anesthetic factors were designated as delayed awakening, hypoxemia, etc. Adverse events were categorized by system: respiratory (e.g., hypoxemia, atelectasis, bronchospasm) (5); cardiovascular (e.g., hypertension, hypotension, arrhythmias); and central nervous system (e.g., postoperative delirium, agitation, somnolence). Surgical factors were designated as postoperative bleeding, as well as “water intoxication” resulting from hysteroscopic procedures. Non-clinical factors were designated as patients who had regained consciousness or stabilized vital signs, yet stayed in the recovery room due to limited transport equipment or staff (22).

### 2.7 Statistical methods

After excluding patients who met the clinical exclusion criteria (items 1-5), Little's MCAR test was conducted on the remaining candidate dataset and revealed no significant deviation from the missing completely at random assumption (*P* > 0.05). We applied complete-case analysis and retained only patients with complete critical PACU records for subsequent analyses; no data imputation was performed.

This study used a 1:3 case-to-control matching ratio, guided by statistical power and cost considerations (23, 24). As a retrospective study, we consecutively enrolled all eligible patients with prolonged PACU stay during the study period, yielding 196 cases in total. To assess sample adequacy for multivariable logistic regression, we applied the events-per-variable (EPV) criterion (25, 26). The final model included seven variables, with an EPV of 28, consistent with recommended thresholds for reliable parameter estimation and model development.

The Shapiro-Wilk test was used to check normality. Continuous variables were summarized as mean ± SD or median (IQR), and categorical variables as frequencies and percentages. Between-group comparisons were performed both within matched sets and among unmatched independent groups. Within matched sets, we used paired t-tests for normally distributed continuous variables, Wilcoxon signed-rank tests for non-normally distributed continuous and ordinal variables, and McNemar's test for binary variables. For unmatched independent groups, we used independent-samples t-tests for continuous variables, Mann-Whitney U tests for non-normally distributed or ordinal variables, and chi-square tests for categorical variables, with Fisher's exact test when expected cell counts were less than 5.

We performed variable selection with L1-penalized LASSO conditional logistic regression (*α* = 1). The optimal penalty *λ* was determined by 10-fold cross-validation following the 1-standard-error (1-SE) rule. Penalty parameters and shrunken coefficient estimates are presented in Supplementary Table 1. Selected variables were then entered into an unpenalized multivariable conditional logistic regression model to obtain final effect estimates. The model's intercept was modified to obtain predicted probabilities. The evaluation of model performance was conducted using discrimination metrics (AUC) and calibration measures (Brier score). We calculated and reported optimism-corrected AUC values to mitigate overfitting bias, and performed decision curve analysis (DCA) to assess clinical net benefit. Internal validation was conducted using bootstrap resampling (1,000 iterations) and 10-fold cross-validation, both stratified by matched sets. To further confirm that multicollinearity did not materially affect the coefficient estimates, we formally assessed variance inflation factors, condition indices, and the correlation matrix based on the final model. Additionally, a sensitivity analysis was performed using unconditional logistic regression to assess the robustness of the findings. Finally, A nomogram and calibration curves were constructed for model visualization and evaluation.

We conducted a *post-hoc* analysis to further investigate the association between preoperative prealbumin level and PLOS in PACU. And the association was visualized using locally weighted scatterplot smoothing (LOESS). Preoperative prealbumin level was categorized into quartiles for the trend test, which was applied to assess statistical significance. All statistical analyses were performed using R (version 4.5.1), and a two-tailed *P* value of < 0.05 was considered to indicate statistical significance.

## 3 Results

### 3.1 Characteristics of patients

From January 2022 to January 2024, a total of 69,123 adult patients who underwent general anesthesia were transferred to the PACU. 58 patients were excluded due to critical PACU records missing, four cases because of planned transfer to ICU, and one case because the surgical procedure was cancelled. Additionally, 348 were excluded because of cardiovascular surgery, 312 for neurosurgical procedures, and 222 patients were those undergoing non-intubated general anesthesia (Figure 1). At last, a total of 68,178 patients were included. 210 prolonged stays in the PACU occurred, and the overall incidence was 0.308%. The etiologies of prolonged stays were distributed as follows: anesthetic factors (93.33%, 196/210). surgical factors (2.86%, 6/210), and non-clinical factors (3.81%, 8/210) (Table 1). Patients in the PLOS group with a prolonged stay due to anesthetic factors were matched. Finally, 784 patients were included in the analysis. Baseline characteristics of the overall study population are summarized in Supplementary Table 2, 3. After case-control matching, the mean age was 69 years, and 60.71% of the participants were female.

**Figure 1:**
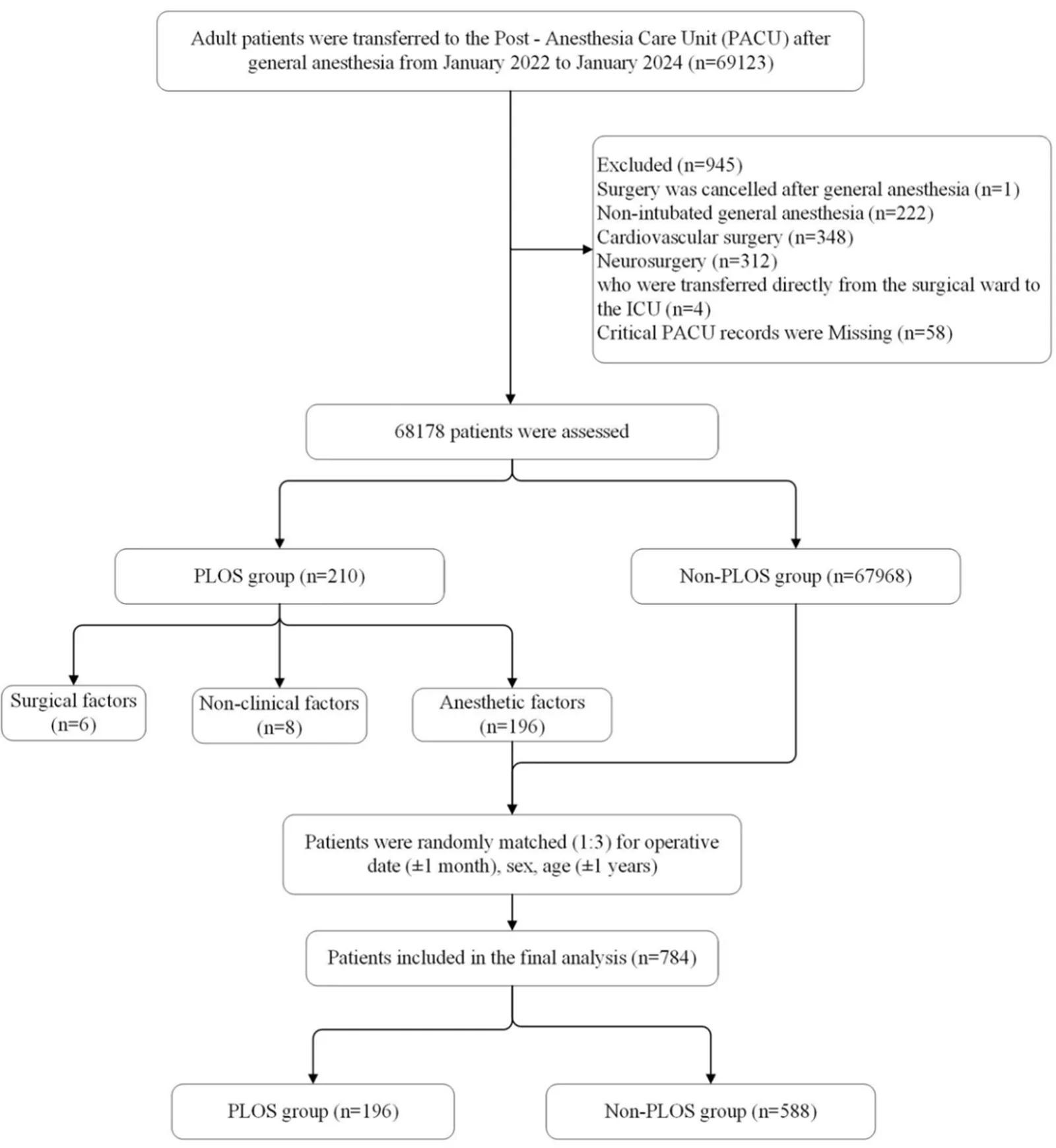
Study flow diagram. This diagram illustrates the process of patient enrollment, screening, and exclusion according to predefined inclusion and exclusion criteria, showing the final number of participants included in the present analysis.

**Table 1:** Reasons and percentage of PLOS in PACU.

Reasons	*n* (%)	Adverse events	*n* (%)
Surgical factors	6 (2.86)	Water Intoxication	1 (0.48)
		Surgical site hemorrhage	5 (2.38)
Non-clinical factors	8 (3.81)	Absence of accompanying personnel for patient transfer	8 (3.81)
Anesthetic factors	196 (93.33)	Delayed awakening	111 (52.86)
		Respiratory adverse events	34 (16.19)
		Cardiovascular adverse events	26 (12.38)
		Central nervous system adverse events	17 (8.10)
		Allergy	4 (1.90)
		Hypothermia	4 (1.90)

PLOS in PACU: Prolonged length of stay in post-anesthesia care unit; Percentages were calculated among all PLOS cases.

### 3.2 Risk factors associated with PLOS in PACU

After LASSO regression, the following 7 variables were selected out: ASA classification, emergency surgery, preoperative prealbumin level, intraoperative blood loss, operative time, cerebrovascular diseases, and surgical classification (Figures 2, 3). Based on the filtered variables, multivariable conditional logistic regression identified emergency surgery, increased intraoperative blood loss, cerebrovascular diseases, longer operative time, and major surgery as potential risk factors for PLOS in PACU. Conversely, a higher preoperative prealbumin level was inversely associated with the risk of PLOS. The magnitude and statistical significance of these factors were detailed in Table 2. Results of the unconditional logistic regression for sensitivity analysis are shown in Supplementary Table 4.

**Figure 2:**
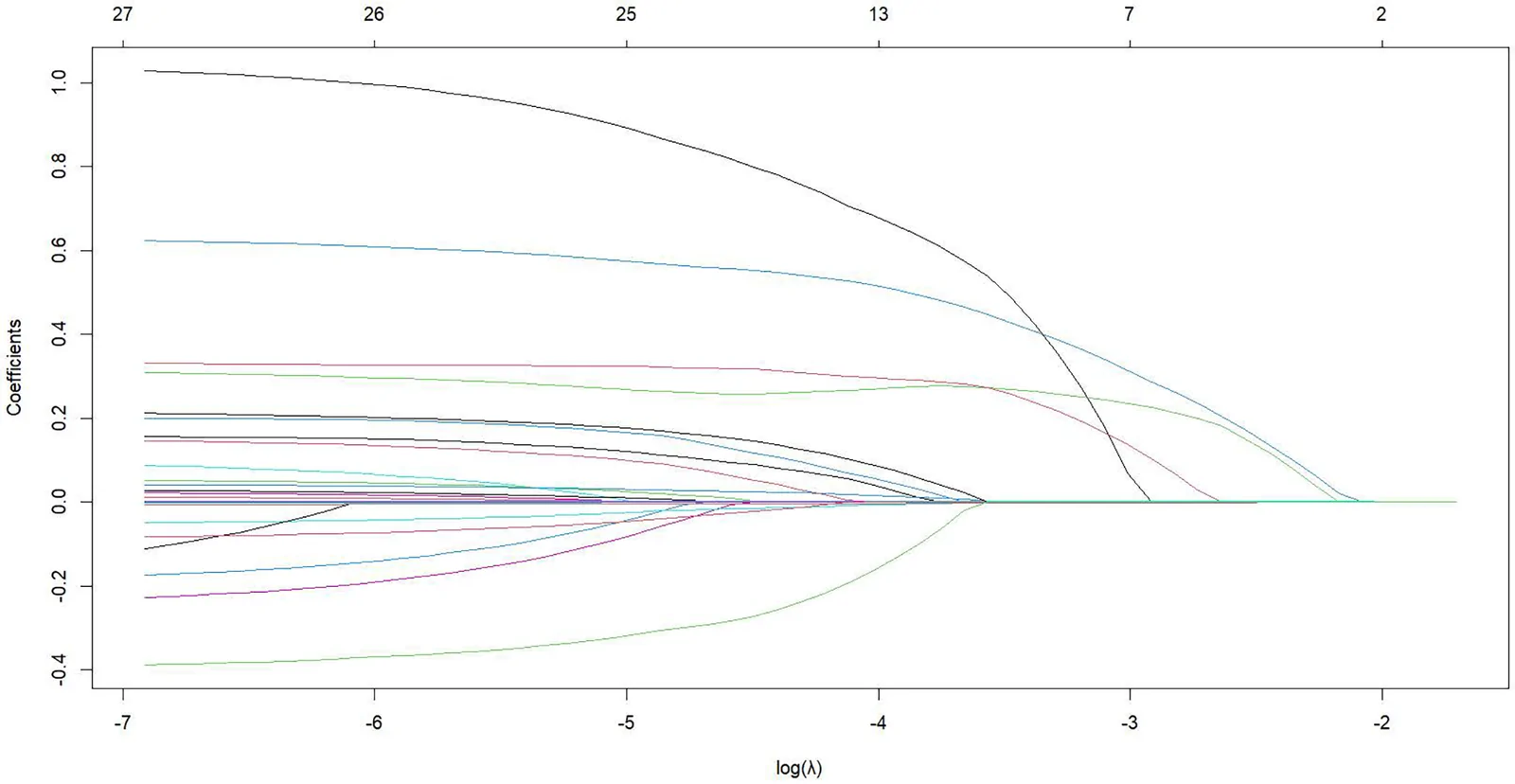
Lasso coefficient path plot. Colored lines represent coefficient trajectories of candidate predictors plotted against log(*λ*). As *λ* increases, certain coefficients shrink toward zero for variable selection.

**Figure 3:**
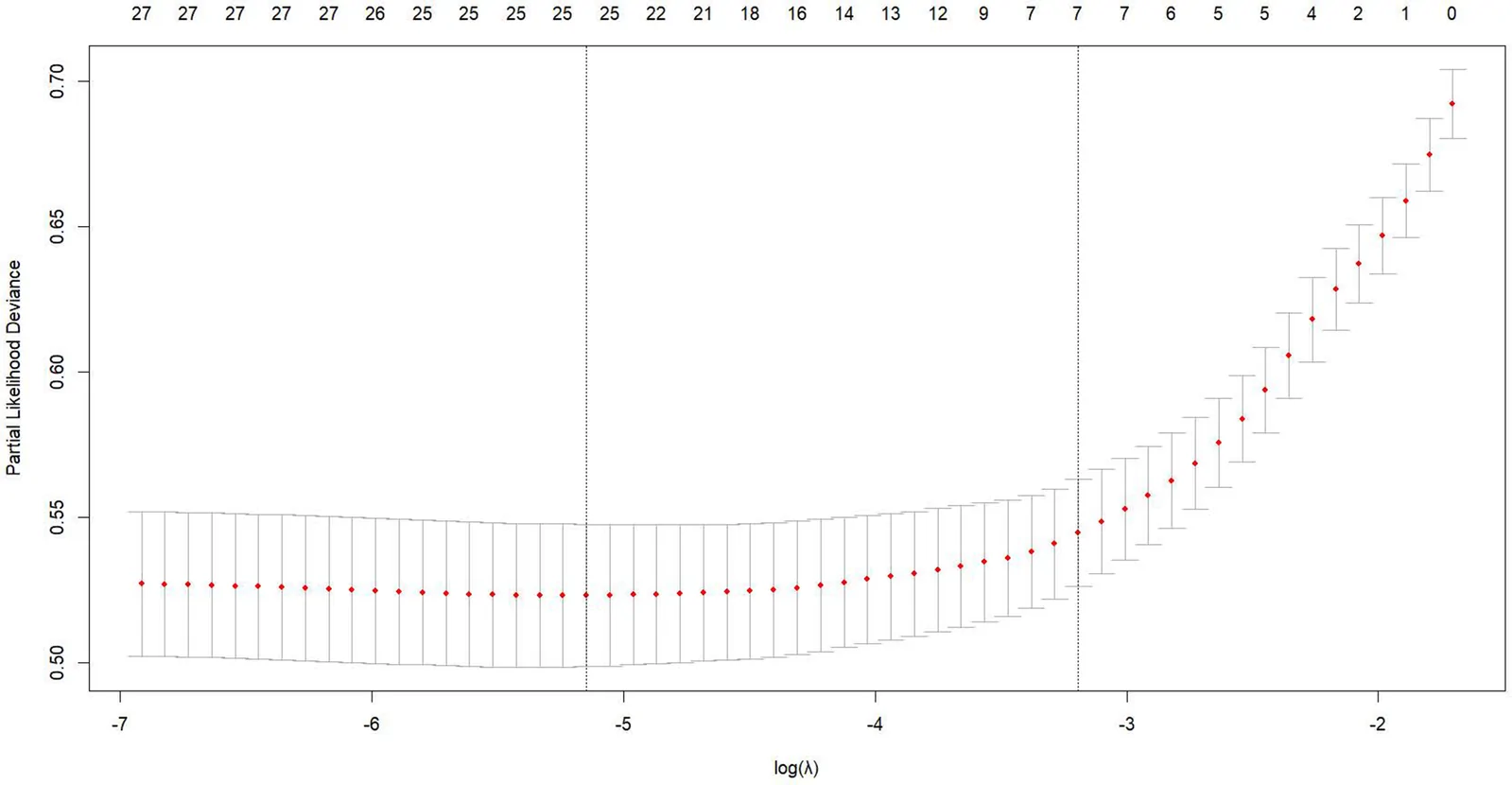
Lasso cross-validation curve. The vertical axis denotes partial likelihood deviance, while the horizontal axis is log(*λ*). Error bars indicate the standard error of deviance. Cross-validation was performed to determine the optimal *λ* for model regularization.

**Table 2:** Multivariable conditional logistic regression results for PLOS in PACU.

Variable	*β*	SE	*Z*-value	*P*-value	OR	95% CI for OR	
Emergency surgery							
No (ref)							
Yes	2.038	0.551	3.698	<0.001	7.678	2.606	22.618
ASA classification							
Ⅰ (ref)							
Ⅱ	0.643	1.044	0.616	0.538	1.901	0.246	14.702
Ⅲ	1.975	1.085	1.821	0.069	7.209	0.860	60.443
Ⅳ	1.968	1.183	1.664	0.096	7.154	0.705	72.642
Preoperative prealbumin level (mg/L)	−0.01	0.002	−4.508	<0.001	0.990	0.986	0.994
Surgical classification							
Minor surgery (Ⅰ) (ref)							
Intermediate surgery (Ⅱ)	0.541	0.458	1.182	0.237	1.718	0.701	4.213
Major surgery (Ⅲ)	1.600	0.516	3.100	0.002	4.954	1.801	13.624
Operative time (min)	0.004	0.002	2.548	0.011	1.004	1.001	1.008
Intraoperative blood loss (mL)	0.002	0.001	2.833	0.005	1.002	1.001	1.004
Cerebrovascular diseases							
No (ref)							
Yes	0.810	0.336	2.407	0.016	2.248	1.162	4.346

Multivariable conditional logistic regression was performed on the matched dataset. β, regression coefficient; SE, standard error; Z-value, Wald test statistic. ref, reference category.

### 3.3 Multicollinearity diagnostics

We assessed multicollinearity in the final conditional logistic regression model using the adjusted generalized variance inflation factor (GVIF). Adjusted GVIF values for all covariates were below 5 in the full-sample analysis, suggesting no substantial multicollinearity at the overall cohort level (Supplementary Table 5). Because conditional logistic regression estimates parameters primarily from within-pair variation, we further assessed within-pair collinearity. All adjusted GVIF values remained below 5 (Supplementary Table 6). The unadjusted GVIF for ASA classification was high but fell to 1.5 after adjustment. When ASA classification was treated as an ordinal variable, ASA classes II and III showed elevated VIF values. This reflects the inherent structural correlation among ordinal levels and suggests limited discriminative contrast between adjacent grades (Supplementary Table 7). The maximum condition index was 8.372 (Supplementary Table 8), and the full-sample Pearson correlation matrix is provided in Supplementary Table 9. Collectively, these diagnostics indicated that the observed correlations did not materially affect the reliability of the coefficient estimates.

### 3.4 Nomogram model: construction, predictive performance, and validation

A nomogram was developed based on the intercept-adjusted multivariable conditional logistic regression model (Figure 4). The calibration curve showed good agreement between the predicted and observed probabilities after bias correction with 500 bootstrap resamples (Figure 5), supported by quantitative metrics: a calibration intercept of −0.059, a slope of 1.010, and an observed-to-expected (O/E) ratio of 0.954. The nomogram achieved an area under the ROC curve (AUC) of 0.893 (95% CI 0.867-0.918) and a Brier score of 0.133 (95% CI 0.114-0.151) (Figure 6). Bootstrap validation (1,000 iterations) generated a mean AUC of 0.894 (95% CI 0.870–0.918) and a mean Brier score of 0.135 (95% CI 0.129–0.155) (Supplementary Figure 1). Ten-fold cross-validation yielded a mean AUC of 0.824 (95% CI 0.762–0.909) and a mean Brier score of 0.140 ± 0.023 (Supplementary Figure 2). The boxplot of AUC values across the three validation methods is shown in Supplementary Figure 3. The optimism-corrected AUC of 0.825 (95% CL 0.801–0.849). Decision curve analysis (Figure 7) showed that the nomogram provided superior net benefit relative to the treat-all or treat-none strategies across clinically meaningful threshold probabilities, supporting its practical utility.

**Figure 4:**
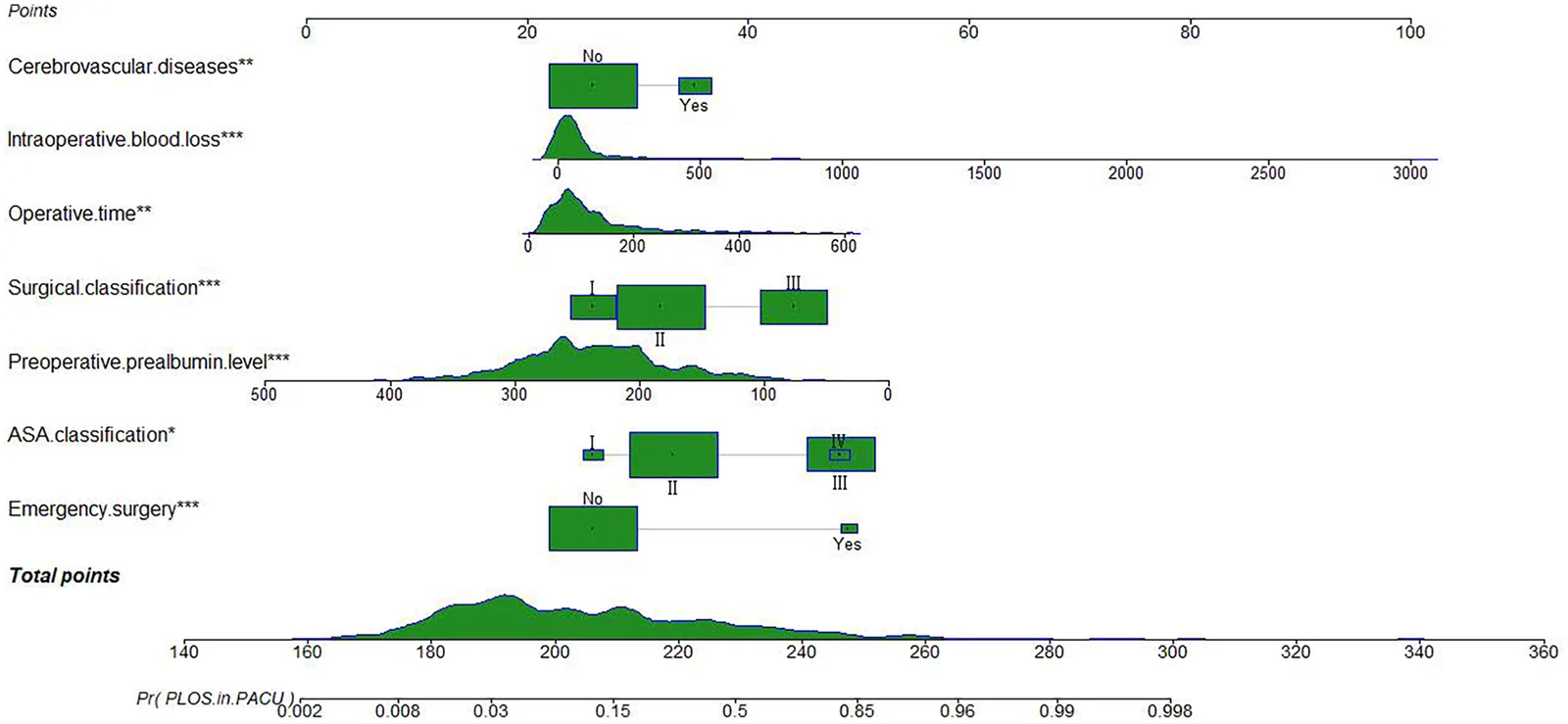
Nomogram prediction model. Scores for each predictor are summed to generate total points, and the total-point scale is converted to the predicted risk probability at the bottom. Asterisks denote significance levels.

**Figure 5:**
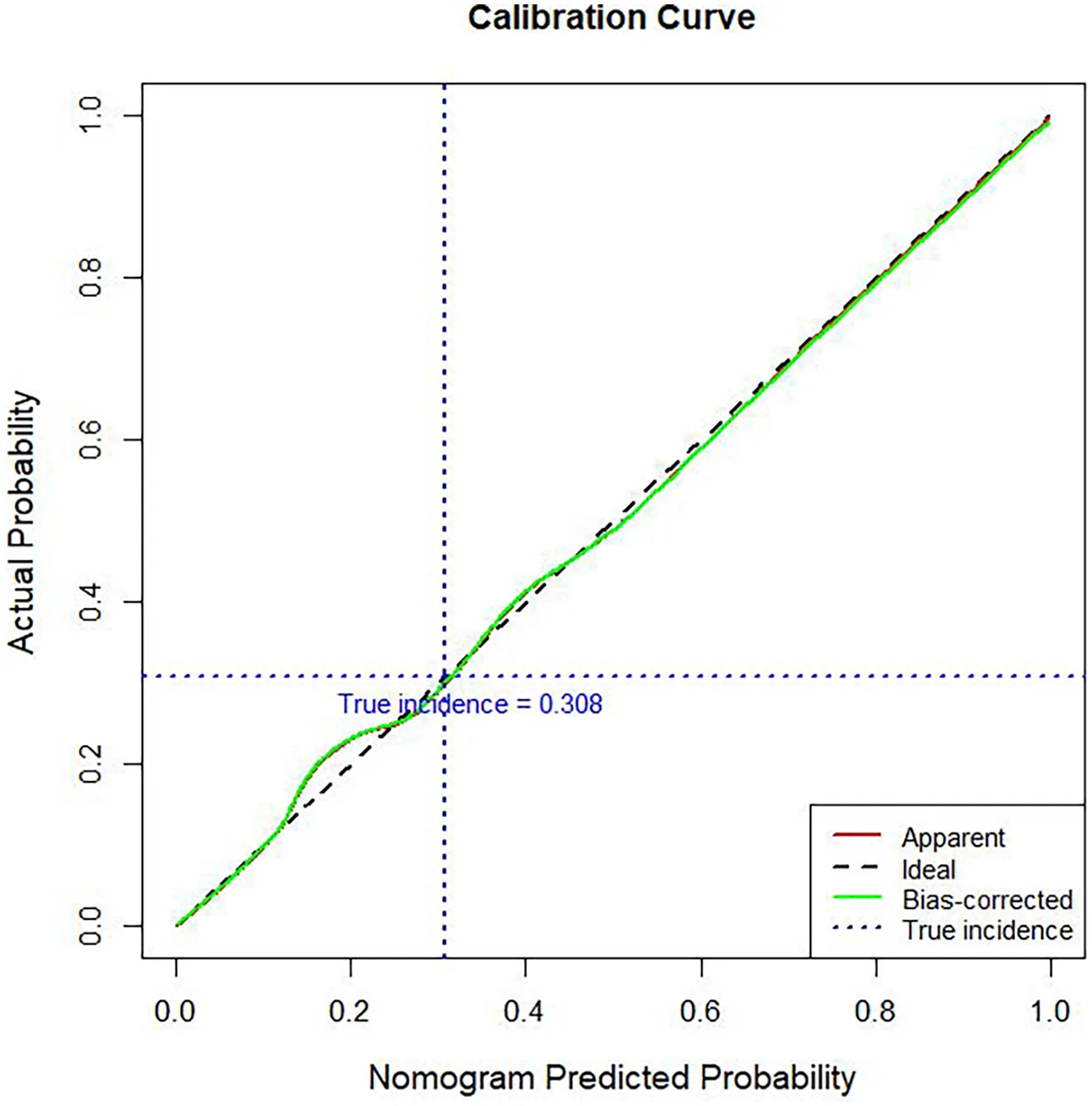
Calibration curve. X-axis: nomogram-predicted probability; Y-axis: actual observed probability. The black dashed line represents ideal prediction. The red line shows apparent performance, while the green line indicates bias-corrected performance via bootstrap. Blue dotted lines indicate the true incidence of the endpoint (0.308%).

**Figure 6:**
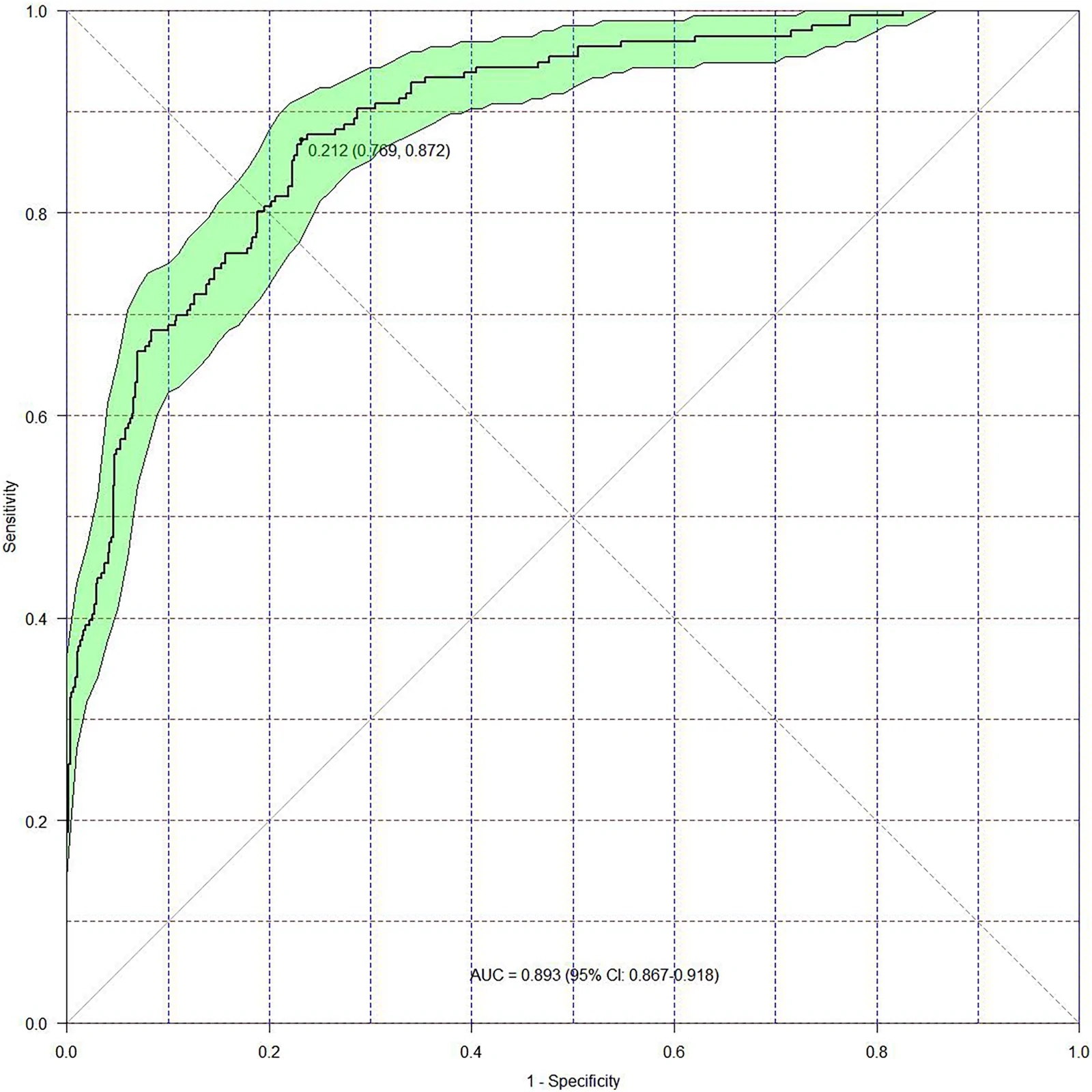
ROC curve for multivariate model. The black step-line represents the ROC curve of the nomogram-based model. The shaded green area indicates the 95% confidence interval of the ROC curve.

**Figure 7:**
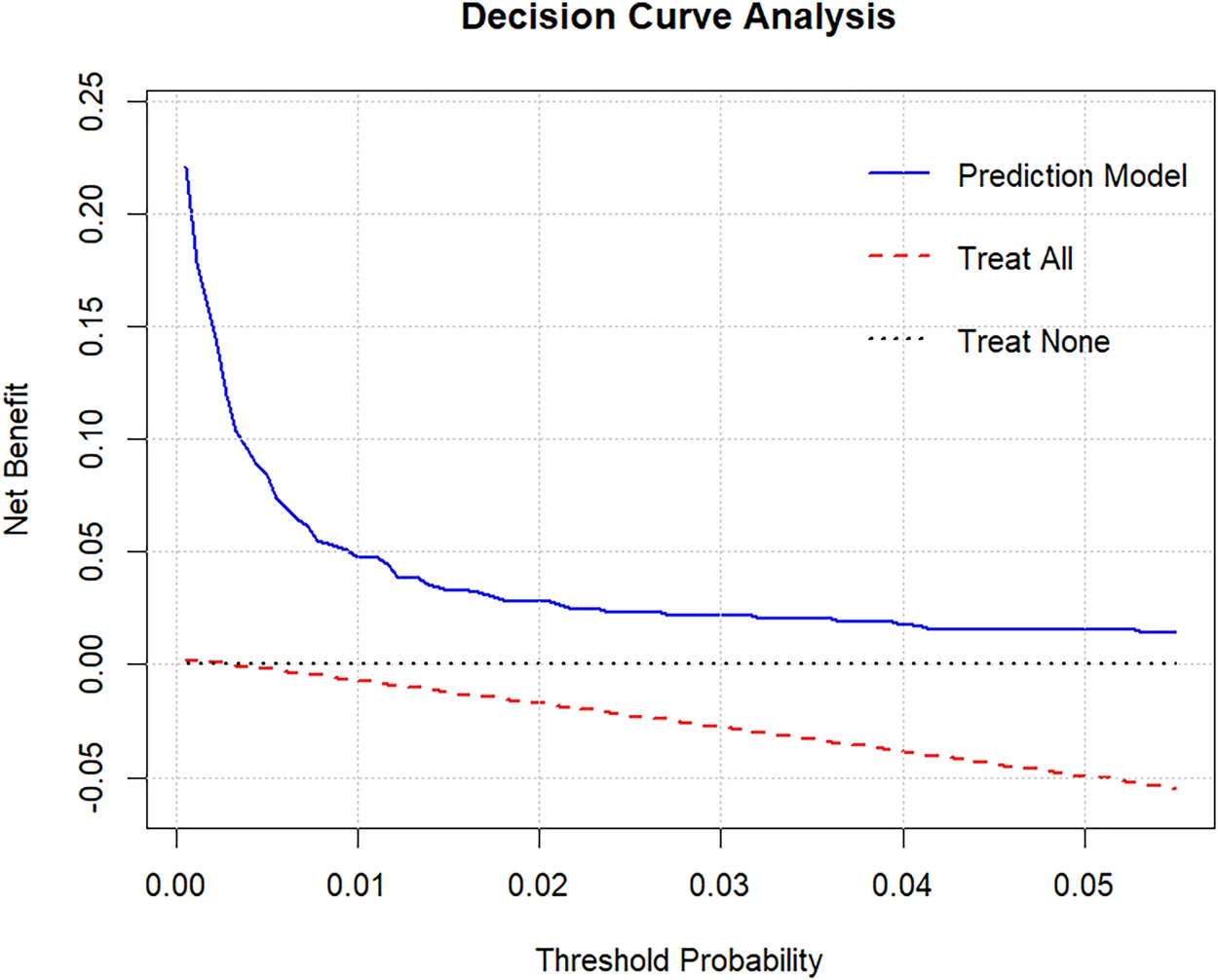
Decision curve analysis for the nomogram predicting prolonged PACU stay. X-axis: threshold probability; Y-axis: net clinical benefit. The blue solid line displays net benefit for the nomogram. The red dashed line and black dotted line represent strategies of treating all patients and treating no patients, respectively.

### 3.5 Association between preoperative prealbumin level and PLOS in PACU

The constructed nomogram demonstrated that preoperative prealbumin level was a significant contributor to the predictive model for PLOS in PACU. The locally weighted scatterplot smoothing (LOESS) curve showed an inverse association: the time of PACU stay was increased as prealbumin level decreased. The relationship was characterized by a rapid descent in stay duration at lower prealbumin levels, with further elevation resulting in a plateau, indicating a point of maximal effect (Figure 8). Furthermore, this non-linear trend was statistically corroborated by a significant trend (*P* for trend < 0.001) across quartiles of preoperative prealbumin level, confirming a progressive reduction in the risk of PLOS in PACU with increasing prealbumin level (Figure 9).

**Figure 8:**
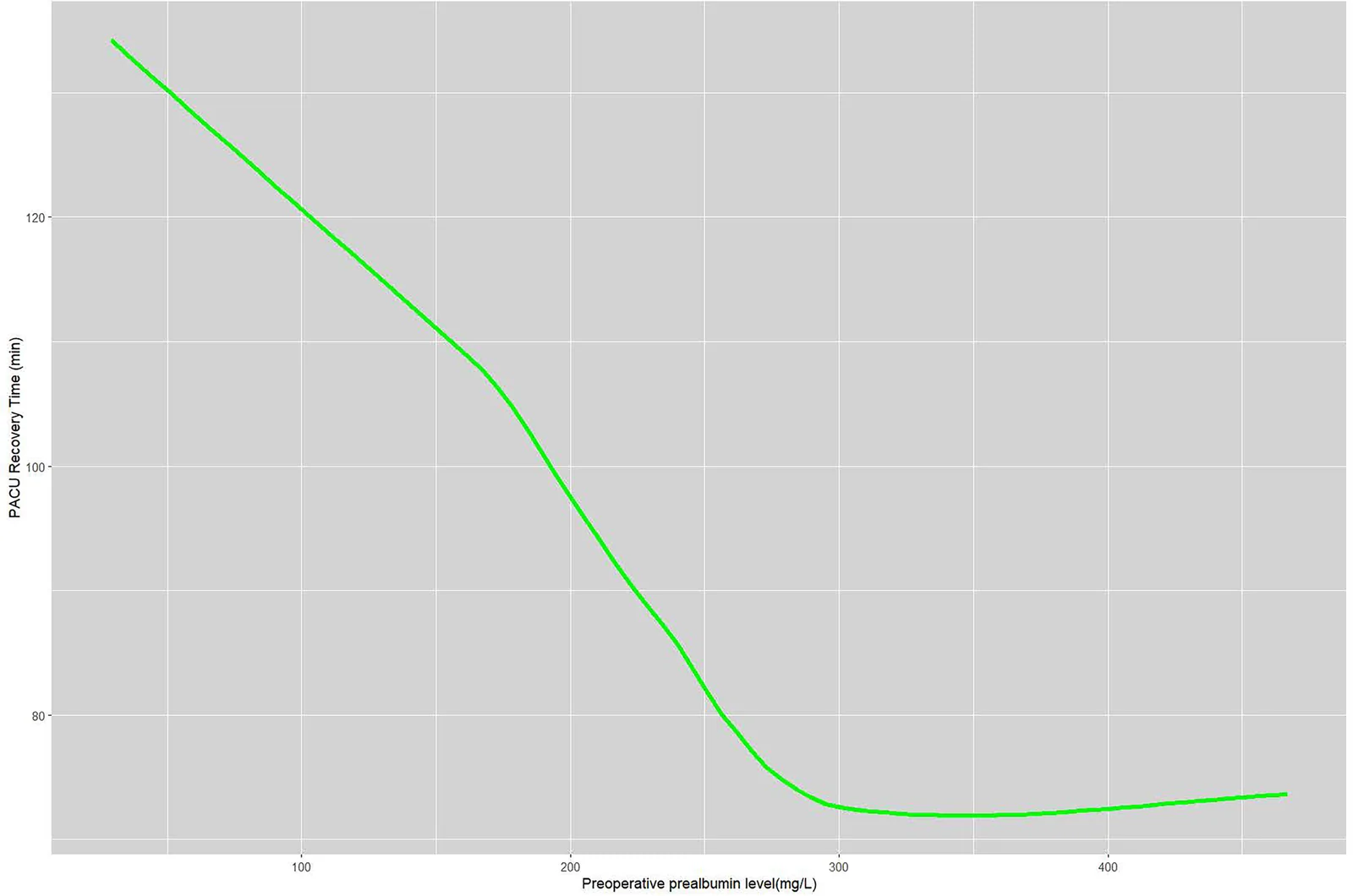
Association between preoperative prealbumin level and PACU recovery time. X-axis: preoperative prealbumin level (mg/L); Y-axis: PACU recovery time (min). The green curve depicts their non-linear association.

**Figure 9:**
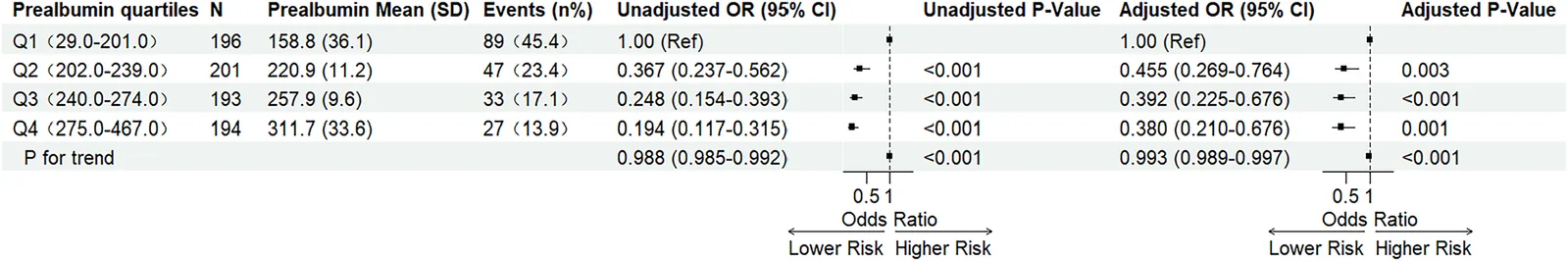
Correlation between preoperative prealbumin level quartiles and PLOS in PACU. 1. Trend analysis utilized the mean preoperative prealbumin level within each quartile as a continuous variable for assessment. 2. The multivariate logistic regression model was adjusted for the following covariates: ASA classification, surgical classification, operative time, emergency surgery, intraoperative blood loss, and cerebrovascular diseases.

## 4 Discussion

The results of this study showed that the rate of PLOS in PACU was 0.308%, excluding neurosurgical and cardiovascular procedures. After adjusting for confounding factors, increased intraoperative blood loss, longer operative time, major surgery, cerebrovascular diseases, and emergency surgery were identified as potential risk factors for PLOS in PACU. A higher preoperative prealbumin level was inversely associated with the risk of PLOS. Further analysis revealed a negative trend between preoperative prealbumin level and the risk of PLOS in PACU.

### 4.1 Differences in prolonged stay rates

In this study, the incidence of PLOS in PACU was 0.308%, which was lower than the previous studies (1, 2, 4, 6, 7, 9–11). This difference from previous findings may be attributed to a key discrepancy in the study population: we excluded patients who underwent neurosurgical procedures, whereas most prior studies did not exclude this patient group. Additionally, differences in the proportion of surgical procedures between hospitals and variations in the definition of PLOS across studies may partially explain the observed disparities in the results.

### 4.2 Collinearity between ASA classification, cerebrovascular diseases, and emergency surgery

Numerous studies show that patients with higher ASA classifications experience longer stays in the PACU (6, 27). Although in this study, it was not independently significant via a Multivariable conditional logistic regression model. This variable showed no collinearity with the other covariates based on prior diagnostic tests. When dummy-coded, ASA classifications II and III yielded VIF values of 10.863 and 11.387, indicating collinearity across ordinal levels and limited discriminative contrast between adjacent levels. From a clinical perspective, ASA classification shows a strong association with emergency surgery and cerebrovascular disease. Patients who undergo emergency surgery are often accompanied by severe comorbidities, while those with cerebrovascular diseases frequently exhibit cognitive impairment perioperatively (28–30). The above factors contribute to a higher ASA classification in these patients (31–34). After these specific clinical indicators were included in the model, ASA classification as a composite measure contributed less to the prediction. Our findings support emergency surgery and cerebrovascular disease as potential risk factors for PLOS.

### 4.3 Interaction among operative time, intraoperative blood loss, and major surgery

This study identified major surgery, longer operative time, and increased intraoperative blood loss as potential risk factors for PLOS in PACU. This was consistent with other studies (12, 16, 35). Major surgery often requires a longer operation time due to its complex surgical procedures, while being accompanied by a higher risk of bleeding (16, 35). Longer operative time also elevates tissue exposure and effusion, which leads to intraoperative hypothermia. These aforementioned factors interact with one another, inducing homeostatic disturbance and hemodynamic instability, and slowing drug metabolism, which in turn affects the recovery in PACU (8, 36, 37).

### 4.4 Association between preoperative prealbumin level and PLOS in PACU

In logistic regression analysis, we first reported that a higher preoperative prealbumin level was inversely associated with the risk of PLOS. The study also revealed that preoperative prealbumin level had a significant non-linear association with PACU length of stay (LOS). In the trend test, we identified a negative trend between preoperative prealbumin level and the risk of PLOS in PACU (*P* for trend, *P* < 0.001). As a serum protein with a half-life of approximately 2.5 days, prealbumin is a sensitive indicator for assessing nutritional status and inflammatory response in surgical patients (38–40). Malnutrition is closely related to frailty and can impair the metabolic response to surgical stress; meanwhile, the inflammatory state caused by surgery can exacerbate pain perception via mediators such as prostaglandins, deplete nutritional reserves, and delay tissue repair (41–45). Persistent low-grade inflammation, metabolic dysregulation, and protein catabolism are prevalent in chronic diseases and frailty, and may contribute to lower prealbumin levels (46–48). Previous studies have indicated that low prealbumin levels were closely associated with prolonged hospital stay, increased postoperative complications, and higher mortality rates (49–51). Thus, a low preoperative prealbumin level is likely a surrogate marker for frailty, inflammation, malnutrition, or chronic illness, rather than an independent causal factor.

### 4.5 Development and clinical utility of a prediction model for PLOS in PACU

Compared with previous prediction models, this study excluded neurosurgical procedures at the design stage, as they are widely recognized as a strong risk factor for prolonged PACU stay (4). We further used a case-control design to adjust for potential confounders and found prealbumin to be a potential novel associated factor. Although ASA classification did not reach statistical significance in our multivariable analysis, it has been a core predictor in previous models (4, 9, 10). Given its established clinical value, we retained it in the final model. The model showed a corrected AUC of 0.825, indicating good discrimination. DCA confirmed a stable net clinical benefit, supporting its clinical utility.

### 4.6 Clinical interpretation of model predictors

When interpreting these findings, it should be noted that several predictors achieved statistical significance, with odds ratios close to unity and of modest magnitude. For retrospective observational studies, statistical significance is not synonymous with clinical relevance. Furthermore, given the low baseline risk, a statistically significant relative association may contribute only limited changes to absolute risk. Accordingly, the odds ratio of any individual predictor should not be overinterpreted. The nomogram developed in this study integrates multiple predictors to generate individualized predicted probabilities, serving as a reference for clinical risk assessment.

### 4.7 Strengths, limitations, and future perspectives of the study

#### 4.7.1 Strengths

This study developed a nomogram to predict PLOS in PACU using routinely collected clinical variables. Neurosurgical patients were excluded, and a case-control matching strategy was implemented to control for established risk factors including age and sex, allowing exploration of other potential risk predictors. We performed rigorous internal validation with stratified bootstrap resampling, 10-fold cross-validation, collinearity diagnostics, and decision-curve analysis to assess the model's discrimination, calibration, and clinical net benefit. This nomogram may help clinicians identify high-risk patients intraoperatively and support individualized risk stratification for PLOS in PACU.

#### 4.7.2 Limitations

First, the generalizability of the model is constrained by multiple factors. As a single-center retrospective study, it presents institutional practice patterns regarding clinical workflows, case-mix, and PACU management protocols. Meanwhile, the matching design excluded well-known predictors including age and sex from the final model. Accordingly, this nomogram derived from matched samples cannot be directly applied to unselected surgical populations prior to external recalibration. In addition, differences in PLOS definitions across studies may limit the comparability of our findings with those of earlier reports. Furthermore, constrained by the completeness of the available medical records, some potential factors may not have been incorporated into the analysis, which may affect the comprehensiveness of the conclusions. Finally, this exploratory study without external validation requires confirmation in independent cohorts.

#### 4.7.3 Future perspectives

Future work should include multicenter external validation to assess the nomogram's predictive performance across diverse case-mix profiles and PACU management protocols. To address the limitation that age and sex were not included in our model due to matching, subsequent studies could build a separate model that includes such established predictors in unselected surgical populations to evaluate their predictive value in general surgical cohorts. In addition, prospective collection of preoperative and postoperative variables not available in the current dataset would permit a more comprehensive model that better captures clinically relevant factors. Validation of the model across different PLOS definitions is also needed to facilitate comparisons across studies using uniform or comparable definitions. Finally, prospective interventional studies are needed to determine whether risk-stratified management based on this nomogram can effectively reduce the incidence of prolonged PACU stay.

## 5 Conclusions

In this matched case-control study, emergency surgery, increased intraoperative blood loss, cerebrovascular diseases, major surgery, and longer operative time were identified as potential risk factors for PLOS. A higher preoperative prealbumin level was associated with a lower risk of prolonged PACU stay, possibly reflecting patients’ preoperative nutritional status, systemic inflammation, chronic illness, or frailty. A nomogram was constructed, though its single-center and exploratory nature requires external validation.

## Data Availability

The data analyzed in this study is subject to the following licenses/restrictions: The datasets used during the current study are not publicly available due to patient privacy restrictions, but are available from the corresponding author on reasonable request, subject to institutional ethics committee approval. Requests to access these datasets should be directed to Xin Wei, kekaiyuan628@126.com.
