# Supplementary material for: Risk factors associated with prolonged stay in the post-anesthesia care unit: a case-control study
Source: Front Med (Lausanne). 2026 Sep 17;13:1906741. doi: 10.3389/fmed.2026.1906741 (PMC13627413; doi:10.3389/fmed.2026.1906741)
Supplement: Supplementary file 1 [file Supplementaryfile1.docx]

**Supplementary Table 1** Variable selection and coefficient shrinkage of LASSO regression under the λ.1se criterion

| Predictors | Coefficient (λ.1se) |
| --- | --- |
| Emergency surgery | 0.273398424 |
| BMI | 0 |
| ASA classification | 0.250771188 |
| Hypertension | 0 |
| Diabetes mellitus | 0 |
| Coronary heart disease | 0 |
| Arrhythmia | 0 |
| Cerebrovascular diseases | 0.193208402 |
| Pulmonary Infection | 0 |
| Serum potassium | 0 |
| Na | 0 |
| Serum chloride | 0 |
| Serum Mg | 0 |
| Ca | 0 |
| Glu | 0 |
| Cr | 0 |
| BUN | 0 |
| ALT | 0 |
| AST | 0 |
| Albumin | 0 |
| Preoperative prealbumin level | -0.002182053 |
| Hemoglobin level | 0 |
| Surgical classification | 0.364090974 |
| Operative time | 0.002344507 |
| Intraoperative blood loss | 0.000120962 |
| Start time of surgery | 0 |
| Analgesic pump | 0 |
| Body Temperature on Admission to PACU | 0 |

The optimal penalty parameter determined by the 1‑standard‑error rule was λ₁ₛₑ = 0.0410601780863096, and the penalty parameter for minimum cross‑validation error was λₘᵢₙ = 0.00582017416684173. A coefficient of zero means the variable was shrunk to zero and excluded from the final LASSO‑logistic model.

**Supplementary Table 2** Baseline characteristics of patients included in the study

| Variable | PLOS (n=210) | Non-PLOS (n=67968) | P-value |
| --- | --- | --- | --- |
| Sex (%) |  |  | 0.301 |
| Male | 81 (38.57) | 28615 (42.10) |  |
| Female | 129 (61.43) | 39353 (57.90) |  |
| Age (years) | 69 (56,76) | 55 (43,66) | <0.001 |
| Elective/emergency surgery (%) |  |  | 0.513 |
| Emergency | 13 (6.19) | 3525 (5.19) |  |
| Elective | 197 (93.81) | 64443 (94.81) |  |
| Hypertension (%) |  |  | <0.001 |
| Yes | 82 (39.05) | 18036 (26.54) |  |
| No | 128 (60.95) | 49932 (73.46) |  |
| Diabetes Mellitus (%) |  |  | 0.043 |
| Yes | 24 (11.43) | 6236 (9.17) |  |
| No | 186 (88.57) | 61732 (90.83) |  |
| Coronary Artery Disease (%) |  |  | 0.142 |
| Yes | 12 (5.71) | 2950 (4.34) |  |
| No | 198 (94.29) | 65018 (95.66) |  |
| Arrhythmia (%) |  |  | 0.023 |
| Yes | 48 (22.86) | 9536 (14.03) |  |
| No | 162 (77.14) | 58432 (85.97) |  |
| Pulmonary Infection (%) |  |  | 0.057 |
| Yes | 68 (32.38) | 20379 (29.98) |  |
| No | 142 (67.62) | 47589 (70.02) |  |
| Cerebrovascular Diseases (%) |  |  | <0.001 |
| Yes | 48 (22.86) | 6009 (8.85) |  |
| No | 162 (77.14) | 61959 (91.15) |  |
| Start time of surgery (%) |  |  | 0.736 |
| 7:30-11:59 (am) | 115 (54.76) | 34633 (50.95) |  |
| 12:00-18:59 (pm) | 82 (39.05) | 28543 (41.99) |  |
| 19:00-7:29 (night) | 13 (6.19) | 4792 (7.06) |  |
| Body temperature at admission to the PACU (%) |  |  | 0.171 |
| 1 = < 36°C | 8 (3.81) | 278 (0.41) |  |
| 2 = 36°C-37.2°C | 198 (94.29) | 66715 (98.16) |  |
| 3 = ﹥ 37.2°C | 4 (1.90) | 975 (1.43) |  |
| BMI (kg/m²) | 22.83 (20.87,25.62) | 23.99 (21.76,26.37) | <0.001 |
| ASA classification (%) |  |  | <0.001 |
| Ⅰ | 6 (2.86) | 3305 (4.86) |  |
| Ⅱ | 86 (40.95) | 33902 (49.89) |  |
| Ⅲ | 102 (48.57) | 29589 (43.53) |  |
| Ⅳ | 16 (7.62) | 1172 (1.72) |  |
| Serum potassium (K⁺) (mmol/L) | 4.12 (3.86,4.39) | 4.09 (3.87,4.32) | 0.129 |
| Serum chloride (Cl⁻) (mmol/L) | 105.70 (103.45,107.41) | 105.60 (103.98,107.30) | 0.447 |
| Serum Mg (mmol/L) | 0.87 (0.82,0.92) | 0.87 (0.82,0.93) | 0.533 |
| Scr (μmol/L) | 58.48 (50.00,71.32) | 58.10 (49.21,70.30) | 0.724 |
| BUN (mmol/L) | 5.87 (4.53,7.04) | 5.79 (4.62,7.24) | 0.735 |
| ALT (U/L) | 20.00 (14.00,29.23) | 20.00 (14.00,28.96) | 0.625 |
| AST (U/L) | 25.00 (19.00,35.00) | 24.00 (19.03,36.00) | 0.052 |
| Albumin (g/L) | 41.53 (37.77,44.26) | 42.55 (39.30,46.32) | <0.001 |
| Preoperative prealbumin level (mg/L) | 208.00 (163.40,247.55) | 248.00 (207.00,286.00) | <0.001 |
| Hemoglobin (g/L) | 127.00 (112.00,137.00) | 131.00 (120.00,143.00) | <0.001 |
| Serum sodium (NA⁺) (mmol/L) | 141.00 (139.00,143.00) | 141.00 (140.00,143.00) | 0.330 |
| Serum calcium (Ca²⁺) (mmol/L) | 2.27 (2.18,2.37) | 2.28 (2.20,2.36) | 0.052 |
| Serum glucose (Glu) (mmol/L) | 5.44 (4.86,6.04) | 5.28 (4.82,5.89) | 0.183 |
| Operative time (min) | 145 (83.50,263.00) | 82.00 (55.00,125.00) | <0.001 |
| Intraoperative blood loss (mL) | 100.00 (20.00,400.00) | 50.00 (10.00,200.00) | <0.001 |
| Surgical classification (%) |  |  | <0.001 |
| Minor surgery (Ⅰ) | 12 (5.71) | 13984 (20.57) |  |
| Intermediate surgery (Ⅱ) | 77 (36.67) | 41462 (61.01) |  |
| Major surgery (Ⅲ) | 121 (57.62) | 12522 (18.42) |  |
| Analgesic pump (%) |  |  | 0.023 |
| Yes | 148 (70.48) | 43515 (64.02) |  |
| No | 62 (29.52) | 24453(35.98) |  |

Results are presented as median (IQR) for continuous variables, and as frequency (percentage) for categorical variables. PACU: Post-anesthesia care unit; PLOS in PACU: Prolonged length of stay in post-anesthesia care unit; Non-PLOS: Non-prolonged length of stay; BMI: Body mass index; ASA: American Society of Anesthesiologists; ALT: Alanine aminotransferase; AST: Aspartate aminotransferase; Scr: Serum creatinine; BUN: Blood urea nitrogen.

**Supplementary Table 3** Baseline characteristics of patients included in the study after case‑control matching

| Variable | Overall population | PLOS (n=196) | Non-PLOS (n=588) | P-value |
| --- | --- | --- | --- | --- |
| Sex (%) |  |  |  | 1.000 |
| Male | 308 (39.29) | 77 (39.29) | 231 (39.29) |  |
| Female | 476 (60.71) | 119 (60.71) | 357 (60.71) |  |
| Age (years) | 69 (57.76) | 69 (57,76) | 69 (57,76) | 0.782 |
| Elective/emergency surgery (%) |  |  |  | 0.052 |
| Emergency | 26(3.32) | 13 (6.63) | 13 (2.21) |  |
| Elective | 758(96.68) | 183 (93.37) | 575 (97.79) |  |
| Hypertension (%) |  |  |  | <0.001 |
| Yes | 249 (31.76) | 81 (41.33) | 168 (28.57) |  |
| No | 535 (68.24) | 115 (58.67) | 420 (71.43) |  |
| Diabetes Mellitus (%) |  |  |  | 0.031 |
| Yes | 68 (8.67) | 22 (11.22) | 46 (7.82) |  |
| No | 716 (91.33) | 174 (88.78) | 542 (92.18) |  |
| Coronary Artery Disease (%) |  |  |  | 0.146 |
| Yes | 31 (3.95) | 12 (6.12) | 19 (3.23) |  |
| No | 753 (96.05) | 184 (93.88) | 569 (96.77) |  |
| Arrhythmia (%) |  |  |  | 0.017 |
| Yes | 129 (16.45) | 48 (24.49) | 81 (13.78) |  |
| No | 655 (83.55) | 148 (75.51) | 507 (86.22) |  |
| Pulmonary Infection (%) |  |  |  | 0.077 |
| Yes | 202 (25.77) | 67 (34.18) | 135 (22.96) |  |
| No | 582 (74.23) | 129 (65.82) | 453 (77.04) |  |
| Cerebrovascular Diseases (%) |  |  |  | <0.001 |
| Yes | 97 (12.37) | 47 (23.98) | 50 (8.50) |  |
| No | 687 (87.63) | 149 (76.02) | 538 (91.50) |  |
| Start time of surgery (%) |  |  |  | 0.848 |
| 7:30-11:59 (am) | 430 (54.85) | 109 (55.61) | 321 (54.59) |  |
| 12:00-18:59 (pm) | 337 (42.98) | 80 (40.82) | 257 (43.71) |  |
| 19:00-7:29 (night) | 17 (2.17) | 7 (3.57) | 10 (1.70) |  |
| Body temperature at admission to the PACU (%) |  |  |  | 0.182 |
| 1 = < 36°C | 8 (1.02) | 7 (3.57) | 1 (0.17) |  |
| 2 = 36°C-37.2°C | 772 (98.47) | 185 (94.39) | 587 (99.83) |  |
| 3 = ﹥ 37.2°C | 4 (0.51) | 4 (2.04) | 0 (0) |  |
| BMI (kg/m²) | 23.66 (21.66,26.04) | 22.88 (20.90,25.77) | 23.94 (22.09,26.12) | 0.001 |
| ASA classification (%) |  |  |  | <0.001 |
| Ⅰ | 23 (2.93) | 2 (1.02) | 21 (3.57) |  |
| Ⅱ | 465 (59.31) | 77 (39.29) | 388 (65.99) |  |
| Ⅲ | 270 (34.44) | 101 (51.53) | 169 (28.74) |  |
| Ⅳ | 26 (3.32) | 16 (8.16) | 10 (1.70) |  |
| Serum potassium (K⁺) (mmol/L) | 4.10 (3.87,4.33) | 4.12 (3.84,4.37) | 4.09 (3.88,4.31) | 0.994 |
| Serum chloride (Cl⁻) (mmol/L) | 105.80 (104.00,107.60) | 105.60 (103.40,107.40) | 105.90 (104.20,107.62) | 0.116 |
| Serum Mg (mmol/L) | 0.87 (0.82,0.92) | 0.87 (0.82,0.92) | 0.87 (0.83,0.92) | 0.468 |
| Scr (μmol/L) | 58.00 (49.22,70.03) | 58.50 (50.00,72.40) | 58.00 (49.00,70.00) | 0.786 |
| BUN (mmol/L) | 5.61 (4.66,6.83) | 5.89 (4.55,7.05) | 5.55 (4.70,6.74) | 0.644 |
| ALT (U/L) | 19.00 (14.00,28.00) | 20 (14.00,29.25) | 19.00 (14.00,27.00) | 0.56 |
| AST (U/L) | 22.00 (19.00,28.00) | 25.00 (19.00,35.00) | 22.00 (18.75,27.00) | 0.048 |
| Albumin (g/L) | 42.20 (39.10,45.20) | 41.60 (37.77,44.20) | 42.50 (39.40,45.40) | <0.001 |
| Preoperative prealbumin level (mg/L) | 239.00 (201.75,274.00) | 208.00 (164.50,249.25) | 249.00 (211.00,281.00) | <0.001 |
| Hemoglobin (g/L) | 128.00 (115.00,138.00) | 126.00 (111.00,136.00) | 129.00 (117.00,139.00) | 0.014 |
| Serum sodium (NA⁺) (mmol/L) | 141.00(140.00,143.00) | 141.00 (139.00,142.00) | 141.00 (140.00,143.00) | 0.003 |
| Serum calcium (Ca²⁺) (mmol/L) | 2.28 (2.19,2.37) | 2.26 (2.17,2.35) | 2.29 (2.20,2.37) | 0.011 |
| Serum glucose (Glu) (mmol/L) | 5.31 (4.75,5.94) | 5.45 (4.85,6.04) | 5.25 (4.74,5.91) | 0.898 |
| Operative time (min) | 92.50 (65.00,145.00) | 161.50 (90.00,276.25) | 85.00 (60.00,123.50) | <0.001 |
| Intraoperative blood loss (mL) | 50.00 (20.00,100.00) | 100.00 (20.00,400.00) | 30.00 (10.00,50.00) | <0.001 |
| Surgical classification (%) |  |  |  | <0.001 |
| Minor surgery (Ⅰ) | 114 (14.54) | 9 (4.59) | 105 (17.86) |  |
| Intermediate surgery (Ⅱ) | 430 (54.85) | 69 (35.20) | 361 (61.39) |  |
| Major surgery (Ⅲ) | 240 (30.61) | 118 (60.20) | 122 (20.75) |  |
| Analgesic pump (%) |  |  |  | <0.001 |
| Yes | 465 (59.31) | 147 (75.00) | 318 (54.08) |  |
| No | 319 (40.69) | 49 (25.00) | 270(45.92) |  |

Baseline characteristics after case‑control matching. Non‑normally distributed continuous variables are presented as medians (25th‑75th percentiles), and categorical variables are shown as n (%). PACU: Post-anesthesia care unit; PLOS in PACU: Prolonged length of stay in post-anesthesia care unit; Non-PLOS: Non-prolonged length of stay; BMI: Body mass index; ASA: American Society of Anesthesiologists; ALT: Alanine aminotransferase; AST: Aspartate aminotransferase; Scr: Serum creatinine; BUN: Blood urea nitrogen.

**Supplementary Table 4** Multivariable analysis results using unconditional logistic regression

| Variable | β | SE | *Z-*value | *P*-value | OR | 95% CI for OR | |
| --- | --- | --- | --- | --- | --- | --- | --- |
| Emergency surgery |  |  |  |  |  |  |  |
| No (ref) |  |  |  |  |  |  |  |
| Yes | 1.730 | 0.457 | 3.788 | <0.001 | 5.640 | 2.294 | 13.933 |
| ASA classification |  |  |  |  |  |  |  |
| Ⅰ (ref) |  |  |  |  |  |  |  |
| Ⅱ | -0.236 | 0.810 | -0.291 | 0.771 | 0.790 | 0.197 | 5.447 |
| Ⅲ | 0.405 | 0.819 | 0.494 | 0.621 | 1.499 | 0.365 | 10.452 |
| Ⅳ | 0.918 | 0.933 | 0.984 | 0.325 | 2.504 | 0.466 | 20.467 |
| Preoperative prealbumin level (mg/L) | -0.007 | 0.002 | -3.785 | <0.001 | 0.993 | 0.990 | 0.997 |
| Surgical classification |  |  |  |  |  |  |  |
| Minor surgery (Ⅰ) (ref) |  |  |  |  |  |  |  |
| Intermediate surgery (Ⅱ) | 0.372 | 0.400 | 0.930 | 0.353 | 1.451 | 0.693 | 3.381 |
| Major surgery (Ⅲ) | 1.232 | 0.438 | 2.813 | 0.005 | 3.429 | 1.510 | 8.536 |
| Operative time (min) | 0.005 | 0.001 | 3.322 | <0.001 | 1.005 | 1.002 | 1.008 |
| Intraoperative blood loss (mL) | 0.002 | 0.001 | 3.202 | 0.001 | 1.002 | 1.001 | 1.003 |
| Cerebrovascular diseases |  |  |  |  |  |  |  |
| No (ref) |  |  |  |  |  |  |  |
| Yes | 0.899 | 0.296 | 3.040 | 0.002 | 2.456 | 1.379 | 4.402 |

Note: Multivariable unconditional logistic regression was performed on the unmatched dataset. β, regression coefficient; SE, standard error; Z‑value, Wald test statistic. ref, reference category.

**Supplementary Table 5** Generalized variance‑inflation factor (GVIF) for assessing multicollinearity among predictor variables

| Variable | GVIF | Df | Adjusted_GVIF |
| --- | --- | --- | --- |
| Emergency surgery | 1.12 | 1 | 1.058 |
| ASA classification | 1.35 | 3 | 1.051 |
| Preoperative prealbumin level | 1.046 | 1 | 1.023 |
| Surgical classification | 1.521 | 2 | 1.11 |
| Operative time | 1.678 | 1 | 1.295 |
| Intraoperative blood loss | 1.478 | 1 | 1.216 |
| Cerebrovascular diseases | 1.317 | 1 | 1.148 |

This table presents generalized variance‑inflation factor (GVIF), degrees of freedom (Df), and adjusted GVIF values for each predictor variable.

**Supplementary Table 6** Group‑level maximum generalized variance‑inflation factor (GVIF) for multicollinearity assessment

| Variable | Df | GVIF group max | Adjusted GVIF |
| --- | --- | --- | --- |
| Emergency surgery | 1 | 1.037 | 1.018 |
| ASA classification | 3 | 11.387 | 1.5 |
| Preoperative prealbumin level | 1 | 1.139 | 1.067 |
| Surgical classification | 2 | 3.276 | 1.345 |
| Operative time | 1 | 2.238 | 1.496 |
| Intraoperative blood loss | 1 | 1.785 | 1.336 |
| Cerebrovascular diseases | 1 | 1.293 | 1.137 |

Note: This table presents group‑level maximum generalized variance‑inflation factor (GVIF), degrees of freedom (Df), and adjusted GVIF values for each predictor variable.

**Supplementary Table 7** individual variance inflation factor (VIF) for each dummy variable at the within-group level

| Dummy Variable | VIF within |
| --- | --- |
| Emergency surgery | 1.037 |
| ASA classification 2 | 10.863 |
| ASA classification 3 | 11.387 |
| ASA classification 4 | 3.031 |
| Preoperative prealbumin level | 1.139 |
| Surgical classification 2 | 2.333 |
| Surgical classification 3 | 3.276 |
| Operative time | 2.238 |
| Intraoperative blood loss | 1.785 |
| Cerebrovascular diseases | 1.293 |

Note: This table presents individual variance‑inflation factor (VIF) values for each dummy variable at the matched‑pair within‑group level.

**Supplementary Table 8** Condition index for multicollinearity diagnosis of the within‑stratum matched model

| Index No | Condition Index |
| --- | --- |
| 1 | 1 |
| 2 | 1.222 |
| 3 | 1.56 |
| 4 | 1.715 |
| 5 | 1.795 |
| 6 | 2.039 |
| 7 | 2.169 |
| 8 | 2.908 |
| 9 | 3.867 |
| 10 | 8.372 |

Note: Index No represents the serial number of the variables; Condition Index is used to diagnose multicollinearity. The maximum condition index was 8.372, indicating no obvious multicollinearity among the included variables.

**Supplementary Table 9** Correlation matrix of independent variables included in the regression model

| Variable | Emergency surgery | ASA.classification2 | ASA.classification3 | ASA.classification4 | Preoperative prealbumin level | Surgicalclassification2 | Surgical.classification3 | Operative time | Intraoperative blood loss | Cerebrovascular diseases |
| --- | --- | --- | --- | --- | --- | --- | --- | --- | --- | --- |
| Emergencysurgery | 1 | -0.05 | 0.046 | 0.045 | -0.055 | 0.068 | -0.092 | -0.042 | -0.046 | -0.026 |
| ASA classification2 | -0.05 | 1 | -0.875 | -0.224 | 0.251 | 0.01 | -0.064 | -0.06 | -0.084 | -0.454 |
| ASA classification3 | 0.046 | -0.875 | 1 | -0.134 | -0.22 | 0.016 | 0.072 | 0.082 | 0.073 | 0.437 |
| ASA classification4 | 0.045 | -0.224 | -0.134 | 1 | -0.172 | -0.047 | 0.078 | 0.048 | 0.096 | 0.147 |
| Preoperative prealbumin level | -0.055 | 0.251 | -0.22 | -0.172 | 1 | 0.037 | -0.12 | -0.18 | -0.244 | -0.134 |
| Surgical classification2 | 0.068 | 0.01 | 0.016 | -0.047 | 0.037 | 1 | -0.732 | -0.393 | -0.287 | 0.03 |
| Surgical classification3 | -0.092 | -0.064 | 0.072 | 0.078 | -0.12 | -0.732 | 1 | 0.607 | 0.429 | 0.053 |
| Operative time | -0.042 | -0.06 | 0.082 | 0.048 | -0.18 | -0.393 | 0.607 | 1 | 0.62 | 0.031 |
| Intraoperative blood loss | -0.046 | -0.084 | 0.073 | 0.096 | -0.244 | -0.287 | 0.429 | 0.62 | 1 | -0.021 |
| Cerebrovascular diseases | -0.026 | -0.454 | 0.437 | 0.147 | -0.134 | 0.03 | 0.053 | 0.031 | -0.021 | 1 |

Note: This table presents correlation‑matrix values for independent variables included in the regression model.
